# Validation of an automatically generated screening score for frailty: the care assessment need (CAN) score

**DOI:** 10.1186/s12877-018-0802-7

**Published:** 2018-05-04

**Authors:** Jorge G. Ruiz, Shivani Priyadarshni, Zubair Rahaman, Kimberly Cabrera, Stuti Dang, Willy M. Valencia, Michael J. Mintzer

**Affiliations:** 1 Veterans’ Successful Aging for Frail Elders (VSAFE) Program, Miami VA Healthcare System Geriatric Research Education and Clinical Center (GRECC), Bruce W. Carter Miami VAMC, 11 GRC, 1201 NW 16th Street, Miami, Florida, 33125 USA; 2 University of Miami Miller School of Medicine, Miami, Florida, USA; 3 Florida International University, Herbert Wertheim College of Medicine, Miami, USA

**Keywords:** Frailty, Screening, Electronic health records, Veterans

## Abstract

**Background:**

Frailty is a state of vulnerability to stressors that is prevalent in older adults and is associated with higher morbidity, mortality and healthcare utilization. Multiple instruments are used to measure frailty; most are time-consuming. The Care Assessment Need (CAN) score is automatically generated from electronic health record data using a statistical model. The methodology for calculation of the CAN score is consistent with the deficit accumulation model of frailty. At a 95 percentile, the CAN score is a predictor of hospitalization and mortality in Veteran populations. The purpose of this study was to validate the CAN score as a screening tool for frailty in primary care.

**Methods:**

This is a cross-sectional, validation study compared the CAN score with a 40-item Frailty Index reference standard based on a comprehensive geriatric assessment. We included community-dwelling male patients over age 65 from an outpatient geriatric medicine clinic. We calculated the sensitivity, specificity, positive predictive value, negative predictive value and diagnostic accuracy of the CAN score.

**Results:**

184 patients over age 65 were included in the study: 97.3% male, 64.2% White, 80.9% non-Hispanic. The CGA-based Frailty Index defined 14.1% as robust, 53.3% as prefrail and 32.6% as frail. For the frail, statistical analysis demonstrated that a CAN score of 55 provides sensitivity, specificity, PPV and NPV of 91.67, 40.32, 42.64 and 90.91% respectively whereas at a score of 95 the sensitivity, specificity, PPV and NPV were 43.33, 88.81, 63.41, 77.78% respectively. Area under the receiver operating characteristics curve was 0.736 (95% CI = .661–.811).

**Conclusion:**

CAN score is a potential screening tool for frailty among older adults; it is generated automatically and provides acceptable diagnostic accuracy. Hence, the CAN score may be a useful tool to primary care providers for detection of frailty in their patient panels.

**Electronic supplementary material:**

The online version of this article (10.1186/s12877-018-0802-7) contains supplementary material, which is available to authorized users.

## Background

Frailty is a state of vulnerability to stressors which may result in higher morbidity, mortality and healthcare utilization in older adults. There are currently two major conceptualization of frailty: the frailty phenotype, which requires the presence of three or more of five components: weight loss, exhaustion, weakness, slowness, and low physical activity [1]; and the deficit accumulation model, which combines symptoms, diseases, conditions and disability into a score called the frailty index (FI) [2]. Such screening can lead to the identification of frail patients in need of diagnostic and management strategies to improve outcomes.

Screening for frailty traditionally involves questionnaires or scales administered in healthcare settings. Several reliable and valid self-report questionnaires have been effectively employed in these settings. They vary by the number of items included, whether administered face-to-face, or self-completed during a clinical encounter or delivered by mail [3, 4]. Health care providers, however, may not have the time to administer these instruments to older individuals; patients may not be willing or able to answer these questions. Clinicians would benefit from an automated tool that assists them in identifying older persons with frailty.

In comparison to the administration of time consuming questionnaires which are subject to interrater variability, automated screenings can have a significant impact on early detection and subsequent evidence-based interventions for frailty in primary care settings. In the US, the Veterans Health Administration has developed and implemented the care assessments needs (CAN) score as a predictive analytic tool that reflects the likelihood of hospitalization or death in an individual patient compared with other patients. The CAN score has been designed to assist primary care providers in patient management and care coordination. The CAN score is generated from VA electronic health record data that includes information on medical conditions, number of diagnoses, vital signs, medications, laboratory tests, use of care coordination resources and overall VA healthcare utilization [5, 6]. The CAN is an objective, efficient, cost-effective and automatically generated score that is ideal as a screening modality. A study of over four million VA patients receiving primary care showed that a CAN score of 95 or higher was a good predictor of hospitalization and death at 90 days and one year with good areas under the receiver operating characteristic (ROC) curve [5, 6]. The methodology for calculation of the CAN score is consistent with the deficit accumulation model of frailty [2] thereby providing a conceptual framework for its use as a valid tool in frailty screening. Using the deficit accumulation model as the conceptual framework, Clegg et al. created an electronic frailty index (eFI) from existing electronic health record data. Similar to the CAN score the eFI showed predictive validity for outcomes of mortality, hospitalization and care home admission. [7]. Currently, the CAN score is available to all VA primary care physicians; the eFI has not been routinely implemented as a tool in clinical practice.

Leveraging the capabilities of electronic health record (EHR) as a data repository for frailty screening may lead to earlier recognition of frail older patients and potentially improve healthcare outcomes for this population. The management of populations of older patients with higher prevalence of frailty substantiates the use of the EHR to identify patients at risk. Automated screening has the potential to bring many more individuals with frailty to the attention of their primary care providers. The purpose of this study is to validate the CAN score as an automated screening tool for frailty in older Veterans.

## Methods

This is a cross-sectional study of community-dwelling Veterans 65 years and older coming to geriatric clinics at a VA Medical Center in the Southeast. Our geriatric clinic is a primary care clinic for adults 65-years and older. The clinic follows the Patient-Centered Medical Home (PCMH) care delivery model whereby patient treatment is coordinated through primary care geriatric medicine physicians including attending physicians and fellows (trainees who have completed internal medicine or family medicine residency training). In the VA Healthcare System, this model is referred to as Patient Aligned Care Teams (PACTs). Study patients included 184 of 371 patients enrolled in the Veterans’ Successful Aging for Frail Elders (VSAFE) clinical demonstration project. Study patients met the following criteria: a CGA and a CAN score were available within a 7-day interval, spoke English, and were seen between July 8, 2016 and June 16, 2017. The CGA served as the reference standard [8–10] and included physical, psychological, functional and social evaluations and when applicable, diagnostic tests. The patients were stratified as robust (FI is ≤0.10), prefrail (FI between 0.10 and 0.20) or frail (FI is ≥0.20) as recommended by Song et al. [11] based on a 40-item frailty index (see Additional file 1) generated from the CGA following the deficit accumulation approach to measuring frailty [12]. Nine geriatric medicine fellows supervised by geriatric medicine staff physicians performed the CGAs during scheduled outpatient clinic appointments. For each patient, we matched the date of the CGA-based frailty index to the CAN-generated admission and death risk at 1 year score generated within a 7-day period. Geriatric medicine fellows and geriatric staff physicians were not aware of the CAN scores on that week. The study was approved by the Miami VA Healthcare System Institutional Review Board as a quality improvement project. Patients were not reimbursed for participation.

Statistical analysis of the data was performed using the SPSS for Apple Macintosh 24 software package (IBM SPSS, Inc., Chicago, IL, USA). Differences among groups on demographic variables, and CAN scores were analyzed by the Levene’s test for homogeneity of variance (*p* = 0.05) and differences between the robust, prefrail and frail groups were analyzed by one-way ANOVA or Welch ANOVA followed by either Tukey’s or the Games-Howell post hoc test when Levene’s values were greater than 0.05 or below 0.05, respectively. Data is presented as mean ± standard deviation. Comparisons of proportions were carried out using the Pearson chi-square test of homogeneity. A Pearson correlation was run to assess the relationship between the CAN score, frailty index, Charlson Comorbidity Index (CCI) and total activities of daily living (ADLs) and instrumental activities of daily living (IADLs) scores (sum of the individual values). Associations were considered significant if *P* < 0.05. Contingency table analysis was utilized to calculate the sensitivity, specificity, positive and negative predictive values (PPV and NPV) at two percentile scores: the values of 95 [5, 6] and 55 - a score that provided a sensitivity over 90% to identify frailty. A desirable screening test would be one that is both highly sensitive and highly specific and, for clinical purposes, displays a high PPV. [13] Receiver Operator Characteristics (ROC) analysis was utilized to assess the CAN score ability to differentiate: robust, prefrail and frail individuals. These analyses were also used to identify the optimal balance between sensitivity and specificity. Area under the curve (AUC) was used to examine improved diagnostic performance. Sample size calculations using an alpha level of 0.05, beta level of 0.10 (90% power), for a minimum expected AUC of 0.70 (moderately accurate test: ≥ 0.7, ≤ 0.9), ratio of frail to non-frail subjects of 3 and null hypothesis value of 0.5 required at least 96 subjects for the present study (MedCalc Statistical Software version 17.9.7, MedCalc Software bvba, Ostend, Belgium; https://www.medcalc.org; 2017).

## Results

The demographic and frailty-related characteristics of the 184 participants were different across the frailty index defined robust, pre-frail and frail groups (Table 1). The frailty index showed that 32.6% of the subjects (*n* = 60) were frail. Compared with robust and prefrail individuals, those with frailty were more likely to be older, have more dependence on ADLS and IADLS, and have higher frailty indexes, and CCI scores. As is common in VA studies, our cohort included few (*n* = 5) women; all scored as frail. There were no differences discovered among Caucasian participants or among non-Hispanics participants.

**Table 1:** Participant characteristics stratified by Frailty status

	Total (*n* = 184, 100%)	Robust (*n* = 26, 14.1%)	Prefrail (*n* = 98, 53.3%)	Frail (*n* = 60, 32.6%)	*p*
Age, mean (SD)	77.34 (8.32)	73.58^a^ (5.78)	77.26^b^ (8.02)	79.10^b^ (9.25)	0.004
Males, n (%)	179 (97.3%)	26^a^ (100%)	98^a^ (100%)	55^b^ (91.7%)	0.005
Caucasian, n (%)	113 (64.2%)	19 (79.2%)	61 (65.5%)	33 (55.9%)	0.394
Not Hispanic, n (%)	148 (80.9%)	23 (88.5%)	77 (79.4%)	48 (80.0%)	0.566
At least 1 ADL dependency, n (%)	44 (23.9%)	0^a^ (0.0%)	11^b^ (11.2%)	33^c^ (55.0%)	< 0.0005
At least 1 IADL dependency, n (%)*	71 (100%)	3^a^ (12.0%)	19^b^ (19.8%)	49^c^ (81.7%)	< 0.0005
Frailty Index, mean (SD)	0.21 (0.13)	0.06^a^ (0.03)	0.16^b^ (0.04)	0.37^c^ (0.09)	< 0.0005
Charlson Comorbidity Index, mean (SD)	6.16 (2.37)	4.2^a^ (1.65)	5.97^b^ (2.23)	7.33^c^ (2.23)	< 0.0005
CAN Score, mean (SD)	67.92 (27.13)	44.04^a^ (28.18)	65.55^b^ (26.1)	82.15^c^ (18.98)	< 0.0005

*Data missing on 3 patients. SD = standard deviation; n = number of participants. Data with different superscript letters are significantly different *p* < 0.05, according to the post hoc ANOVA statistical analysis for continuous variables and chi square for categorical variables. The column means test table assigns a superscript letter (a, b or c) to the robust, prefrail and frail groups. If a pair of values is significantly different, the values have different subscript letters assigned to them. If a pair of values are not significantly different, the values will have the same superscript letters assigned to them. Data without superscripts is not significantly different between robust, prefrail and frail groups

**Fig. 1:**
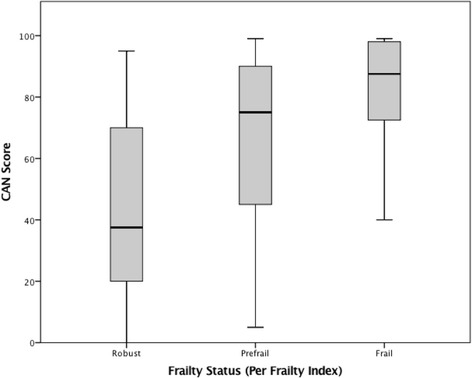
Correlation of CAN Score and Frailty Index in Same 7-Day Interval. The shaded box shows the interquartile range. The ends of the box show the first quartile (the 25% mark) and the third quartile (the 75% mark). The lower part of the chart at the end of the lower “whisker” is the minimum and the upper is the maximum. The median is represented by the horizontal bar in the center of the box

The CAN score was statistically significantly different between robust, prefrail and frail groups [Welch’s F(2, 65.526) = 24.089, *p* <  0.0005]. The mean CAN score increased from the robust (44.04, SD = 28.179, 95% CI [32.66–55.42]), to the prefrail (65.55, SD = 26.1, 95% CI [60.32–70.78]) and frail (82.15, SD = 18.983, 95% CI [77.25–87.05]) groups. The box plot shows that the CAN score increases from the robust to the prefrail and the frail groups (Fig. 1). Post hoc analysis revealed the increase from robust to prefrail (21.51, 95% CI [6.57–36.46]) was statistically significant (*p* = 0.003), as well as the increase from prefrail to frail (16.599, 95%CI [8.08–25.12], *p* < 0.0005. ROC curves were for the frail versus the combined robust and prefrail groups showed an area under the curve (AUC) of 0.736 (95% CI = .661–.811).

Table 2 shows the variations in specificity and sensitivity by 95^th^ and 55^th^ percentiles for patients with frailty. At the 95th percentile, specificity is higher than sensitivity; PPV and NPV are both high. At the 55^th^ percentile, sensitivity is higher than specificity; NPV is dramatically higher than PPV. In clinical medicine, the PPV, a percentage based on the fraction: true positives / (true positives + false positives), may be most helpful. At the 95th percentile, approximately 2 of 3 patients would be frail; at the 55^th^ percentile, approximately 2 of 5 older patients would be frail. We found moderate positive correlations between the CAN score and both the frailty index (*r* = .454, *p* = 0.01) and the CCI (*r* = .335, *p* <  0.0005). The correlations with the total ADLS score (*r* = .184, *p* = 0.05) and IADLS total score (*r* = .287, *p* = 0.01) were positive but small.

**Table 2:** Sensitivity, specificity, PPV and NPV of the CAN score for Frailty screening

CAN Score cut-off	Sensitivity (95% CI)	Specificity (95% CI)	PPV (95% CI)	NPV (95% CI)
≥ 55^th^ percentile	91.67% (81.61–97.24)	40.42% (31.6–49.51)	42.64% (38.69–46.68)	90.91% (80.79–95.56)
≥ 95^th^ percentile	43.33% (30.59–56.76)	88.81% (82.21–93.60)	63.41% (49.81–75.17)	77.78% (73.56–81.49)

*CI* Confident Intervals, *PPV* Positive predictive value, *NPV* Negative predictive value

## Discussion

Two existing automated tools, the CAN score and the eFI are good predictors of health care utilization and mortality [5, 14]. However, of the two, only the eFI has been validated as a frailty diagnostic tool [14]. The fact that the CAN score is currently implemented in the largest integrated healthcare system in the US represents an excellent opportunity to test its ability to screen for frailty in a population of older adults. The use of the CAN score as a system-wide automated screening program to identify frailty in older adults will allow physicians to direct evidence-based interventions to improve clinical outcomes to those most in need. Compared to a CGA-based frailty index used as the reference standard for diagnosing frailty, this study provides validation that the automated CAN score can differentiate frail and non-frail patients. This automated approach can serve as an initial frailty assessment for screening patient panels in primary care settings.

Individuals with higher CAN scores were more likely to be frail than those with lower scores. Concurrent validity is evident by the significant positive correlations obtained between the two CAN scores used in this study and the CGA-based frailty index scores. ROC analysis demonstrated that the AUC was fair and able to provide frailty cut-off scores with high sensitivity and NPV at a 55^th^ percentile and high specificity and PPV at a 95^th^ percentile. To our knowledge, this is the first time that the CAN score has been validated for frailty screening.

Obstacles to early detection of frailty are multiple, including a lack of familiarity with the condition, unrecognized symptoms and signs, denial or lack of awareness of the condition, interpretation of symptoms as part of the normal aging process by patients, family members and primary care clinicians, and time constraints to conduct appropriate evaluations in busy primary care practices. Furthermore, screening for frailty should be an easy, quick, and low-cost process able to detect most patients with the condition.

Currently in primary care settings, a frailty screening test with high sensitivity (over 90%) may be more desirable than high specificity, as it would minimize the risk of missing a patient with frailty - a serious medical condition for which an intervention is available. It is, however, important to realize that CGA-based frailty index, used to confirm the results of frailty screening, is time consuming and often the expertise to administer CGA-based frailty index is unavailable. A high specificity would be desirable to minimize the number of false positives which can burden already strained geriatric services. In this study, the CAN score at a 95th percentile demonstrated a low sensitivity but a high specificity for identifying frailty. Healthcare organizations with limited access to geriatric expertise and resources may decide that a high specificity CAN score of 95 will ensure the cost-effectiveness of the frailty screening program. Under this screening strategy, most individuals without frailty will not be unnecessarily referred for CGA. Conversely, organizations with available geriatric services may prefer a CAN score of 55 corresponding to a higher sensitivity but a lower specificity for the identification of frailty. The CAN score sensitivity at 55 is superior to most frailty questionnaires and scales [15–17] and comparable to other performance based measures (e.g., gait speed, short physical performance battery) [3]; however, the AUC and specificity are lower than most instruments [3, 15, 17]. The PPV of 42.6% (57.4% false positives) will result in a number of patients being referred for a CGA-based frailty index who will not test positive for frailty. This high false positive rate is comparable with widely used screening tests such as the prostate specific antigen (PSA) for prostate cancer of around 30% [18–20], a condition with similar prevalence to that of frailty in the older population, and superior to the under 20% PPV of low dose CT for lung cancer [21, 22], a condition with a lower prevalence. Importantly, unlike those tests, the CAN score does not result in the need for invasive testing. The CGA-based frailty index requires only an office visit and may have the additional benefit of uncovering other unrecognized geriatric syndromes or conditions.

Clearly, there are resource implications depending on the cut-off score used.

At the 95^th^ percentile most would be frail; however, the absolute numbers would likely be less than using a 55^th^ percentile. Furthermore, it is very likely that many in the 95^th^ percentile, even those not formally diagnosed with frailty, would already be receiving comprehensive care for complex illness in other clinical programs. If resources are limited, targeting patients in the 95^th^ percentile who are not already receiving specialized care is a reasonable alternative to initiate a frailty screening program. If resources are available to perform a CGA-based frailty assessment on all individuals with a CAN of 55, a frailty assessment program, that also excludes those receiving specialized care, could be initiated. Identifying 4 of 10 patients with true frailty is patient-centered, quality of life focused, and will also include the reward of additional organizational cost reductions. Another strategy to address the high false positive rates may be repeat testing over time [23]. Contrasting with the difficulties of administering questionnaires or conducting performance-based tests, repeated testing with the CAN score becomes a feasible and more cost-effective approach. As frailty may be a reversible [24, 25], having several data points at which the CAN score is generated over time would improve the accuracy of screening. This approach will require further study.

A major strength of this work is the widespread use of the validated CGA as a clinical tool based on millions of patient visits. Another strength of our study is the relatively large sample size and the completeness of our data. Although initially not considered in the design and development of this study, the CAN score fits within the deficit accumulation paradigm of frailty which supports construct validity between the tools. Limitations of our study was the inclusion of mostly male patients enrolled in a geriatric medicine clinic. Older patients in other primary care clinics with a larger number of older women may be different from this sample. Although the number of participants appears low, the sample exceeds the calculated minimum number of participants needed.

## Conclusions

The CAN score has validity as a screening tool for detection of frailty and warrants further investigation regarding its applicability in primary care settings. The CAN score may help clinicians recognize frailty sooner and implement strategies for improving patient outcomes in older patients with frailty. Further studies are needed to help identify and refine new data or measurements that will improve the screening accuracy of clinical tools to find older adults with frailty.

## Additional file

Additional file 1: **Appendix 1.** Care Assessment Need Score Data Elements. **Appendix 2.** Frailty Index Data Elements. (DOCX 17 kb)

## Abbreviations

ADLS: Activities of daily living
ANOVA: Analysis of Variance
AUC: Area under the curve.
CAN: Care Assessment Need
CGA: Comprehensive Geriatric Assessment
EHR: Electronic health record
IADLS: Instrumental Activities of daily living
NPV: Negative predictive value
PPV: Positive predictive value
ROC: Receiver Operator Characteristics
VA: Veterans Affairs
VSAFE: Veterans’ Successful Aging for Frail Elders
