# Supplementary material for: Validation of an automatically generated screening score for frailty: the care assessment need (CAN) score
Source: BMC Geriatr. 2018 May 4;18:106. doi: 10.1186/s12877-018-0802-7 (PMC5935952; doi:10.1186/s12877-018-0802-7)
Supplement: Supplementary file 1 — Appendix 1. Care Assessment Need Score Data Elements. Appendix 2. Frailty Index Data Elements. (DOCX 17 kb) [file 12877_2018_802_MOESM1_ESM.docx]

**Supplementary Material**

**Appendix 1: Care Assessment Need Score Data Elements**

1. Demographics:

Age (≥65)

Marital status

Service connection more than 50%*

Sex

1. Chronic Illness:

Atrial fibrillation

Atherosclerotic Peripheral Vascular Disease.

**COPD**

Dementia

Depression

Deyo-Charlson comorbidity index

**Diabetes**

Functional disease

**Hepatocellular carcinoma**

Hypertension

Liver disease

Malnutrition

**Metastatic Cancer**

**Myocardial infarction/Unstable angina/CABG**

Pneumonia

Psychiatric disease

PTSD

Valvular disease

**Renal Failure**

Respiratory failure

**Stroke**

Trauma

1. Utilization:

Bed days of care (1-10 vs 0)

Cardiology visits

ER visits (>1 first year)

Mental health visits

Number of providers (>3)

Outpatient visits (>4)

Other visits (>3)

Primary care visits (>1)

Pulmonary visits

Recent Admission

1. Vital Signs:

BMI (<25)

Heart rate (>85)

Systolic & Diastolic BP

Respiratory rate (≥20)

1. Pharmacy:

ACE inhibitors/ARB

Alpha-blocker

Anti-depressants

Antiplatelet drugs

Antipsychotics

Benzodiazepine

Beta-blockers

Bumetanide/ Torsemide

Calcium channel blockers

Digoxin

Furosemide

Insulin

Lipid lowering drugs

Metformin

Metolazone

Nitrate-long acting

NSAIDS

Nebulized drugs

Opioids

Potassium sparing diuretic

PPAR-gamma agonists

Oral steroids

Thiazides

Warfarin

1. Interactions:

18 drug interaction terms

____________________________________________________________________________

Data elements in bold text overlap with those in the FRAIL scale.

* Disability compensation is a monetary benefit paid to Veterans who are determined by VA to be disabled by an injury or illness that was incurred or aggravated during active military service. These disabilities are considered to be service connected.

**Appendix 2: Frailty Index Data Elements**

1. Help Bathing (Yes, No)
2. Help Dressing (Yes, No)
3. Help Using Toilet (Yes, No)
4. Help transferring (Yes, No)
5. Incontinent (Yes, No)
6. Help Feeding (Yes, No)
7. Help using Telephone (Yes, No)
8. Help Shopping (Yes, No)
9. Help with Food Preparation (Yes, No)
10. Help with laundry (Yes, No)
11. Help with Housekeeping (Yes, No)
12. Help taking Medication (Yes, No)
13. Help with Finances (Yes, No)
14. Lost more than 10 lbs. in last year (Yes, No)
15. Hypertension (Yes, No)
16. Heart attack (Yes, No)
17. CHF (Yes, No)
18. Stroke (Yes, No)
19. Cancer (Yes, No)
20. Diabetes (Yes, No)
21. Arthritis (Yes, No)
22. Chronic Lung Disease (Yes, No)
23. PTSD (Yes, No)
24. Schizophrenia (Yes, No)
25. Anxiety (Yes, No)
26. Bipolar (Yes, No)
27. Depression (Yes, No)
28. BP, systolic (mmHg) (<90, >140, 90-140)
29. BP, diastolic (mmHg) (<60, >90, 60-90)
30. MoCA (<26, ≥26)
31. Gait Speed (<1 m/sec, ≥1 m/sec)
32. Body mass index, BMI (<18 or >31, 18-31)
33. Number of Medications (≥ 9, <9)
34. Lives alone (Yes, No)
35. Smoking (Yes, No)
36. Alcohol Abuse (Yes, No)
37. Substance Abuse (Yes, No)
38. Falls in last year (Yes, No)
39. African American (Yes, No)
40. Homeless (Yes, No)

Abnormal values and “Yes” responses = 1 point

Normal values and “No” responses = 0 points.
